# Supplementary material for: DNA Topoisomerase I differentially modulates R-loops across the human genome
Source: Genome Biol. 2018 Jul 30;19:100. doi: 10.1186/s13059-018-1478-1 (PMC6066927; doi:10.1186/s13059-018-1478-1)
Supplement: Supplementary file 1 — Figure S1. A. Quantitation of R loop signal from dot blot shown in Fig. 1. Error bars are SE of three independent experiments. A representative DNA loading control is shown at right after agarose gel electrophoresis. B. Dot blot and relative quantitation to measure R loop loads in genomic DNA from control or Top1 depleted cells, with and without RNase H pre-treatment. C. Dot blot depicting change of global R-loop signal 4, 5, and 6 days after initial Top1 depletion. Samples are indicated at left. D. Distribution of DRIPc signal (right) across genomic compartments compared to expected distribution in the genome (left). Color codes are as described in Fig. 1c. E. Genome browser screenshot illustrating signal “spreading” in the Top1-depleted samples. The R-loop signal for the (+) strand is shown in red. The high-sensitivity peak calls developed to capture signal spreading are found below each track. F. XY plot of average log(10) count-normalized DRIPc-seq signals for control samples (scramble – x axis) and top1-depleted samples (y-axis). G. Validation by DRIP-qPCR of R-loop gain and loss loci identified by DRIPc-seq. Error bars are SE of three independent experiments. H. Validation by DRIP-qPCR R-loop gain (left) and loss (right) upon depletion of Top1 by a second, independent siRNA. The inset above shows a Western blot verifying Top1 depletion. I. Validation by DRIPqPCR of RLL and RLG loci identified by DRIPc-seq 5 and 6 days after Top1 depletion. Error bars represent SE of 2 independent experiments. J. Validation of R-loop loss and gain loci identified by DRIPc-seq using S9.6-independent DRIVE-pPCR method. The average and standard deviation of two independent replicates is shown. The RPL13A locus represents an invariant control. K. DRIPqPCR analysis of R-loop formation over the 5’ ETS and 28S rDNA regions. Results are average of 3 independent experiments shown with standard deviation. RNase A and RNase H pre-treatments are indicated below. L. DRIPqPCR analysis of R-l [file 13059_2018_1478_MOESM1_ESM.pdf]

Figure S1

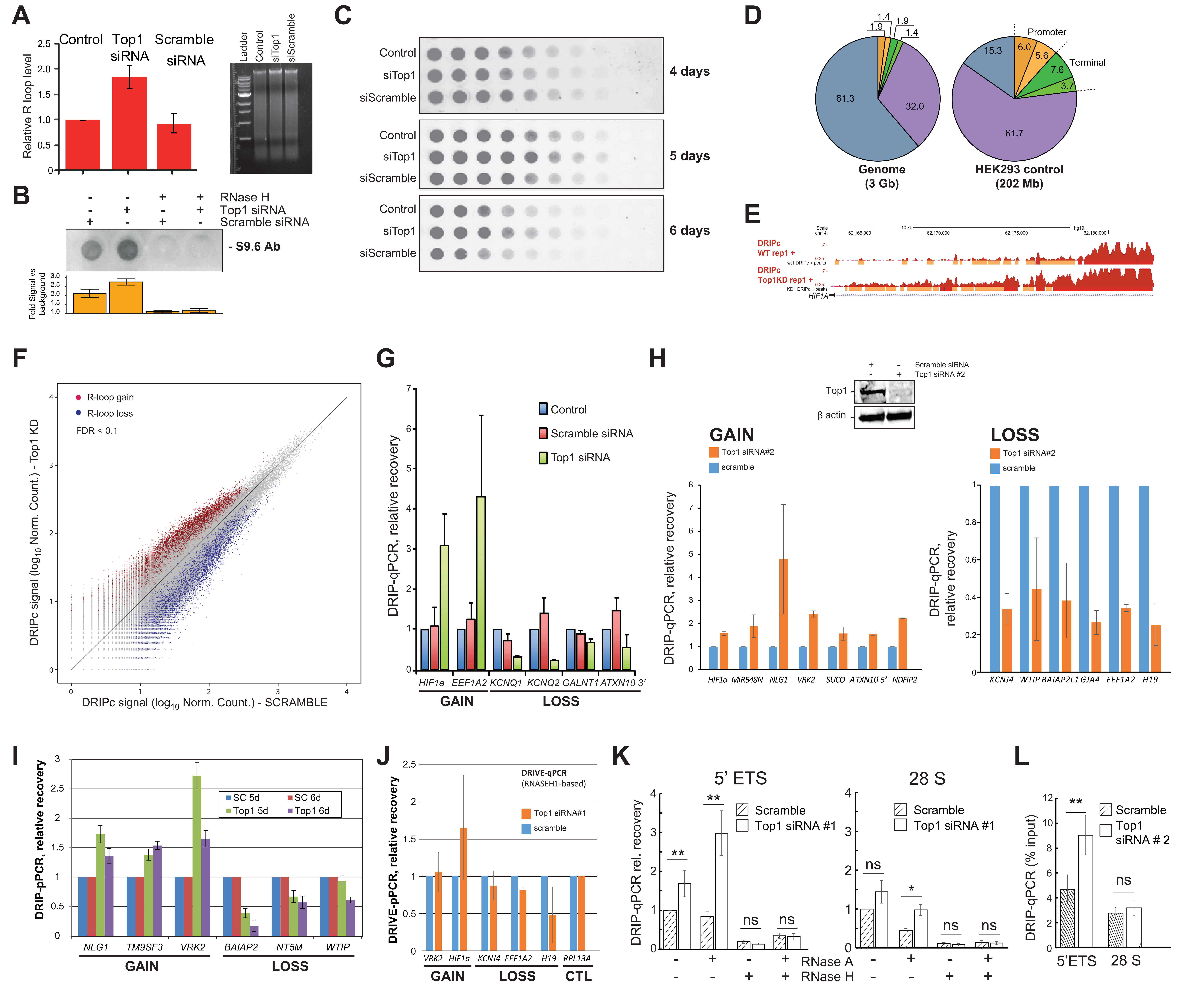

A
Control
Top1 siRNA
Scramble siRNA
Relative R loop level
Ladder
Control
siTop1
siScramble
B
RNase H
Top1 siRNA
Scramble siRNA
- S9.6 Ab
Fold Signal vs background
C
Control
siTop1
siScramble
4 days
5 days
6 days
D
61.3
32.0
15.3
6.0
5.6
7.6
3.7
61.7
Promoter
Terminal
Genome (3 Gb)
HEK293 control (202 Mb)
E
DRIPc WT rep1 +
DRIPc Top1KD rep1 +
HIF1A
F
R-loop gain
R-loop loss
FDR < 0.1
DRIPc signal (log10 Norm. Count.) - Top1 KD
DRIPc signal (log10 Norm. Count.) - SCRAMBLE
G
Control
Scramble siRNA
Top1 siRNA
DRIP-qPCR, relative recovery
HIF1a
EEF1A2
KCNQ1
KCNQ2
GALNT1
ATXN10 3'
GAIN
LOSS
H
Scramble siRNA
Top1 siRNA #2
Top1
β actin
GAIN
LOSS
Top1 siRNA#2
scramble
DRIP-qPCR, relative recovery
HIF1a
MIR548N
NLG1
VRK2
SUCO
ATXN10 5'
NDFIP2
KCNJ4
WTIP
BAIAP2L1
GJA4
EEF1A2
H19
I
SC 5d
SC 6d
Top1 5d
Top1 6d
DRIP-pPCR, relative recovery
NLG1
TM9SF3
VRK2
BAIAP2
NT5M
WTIP
GAIN
LOSS
J
DRIVE-qPCR
(RNASEH1-based)
Top1 siRNA#1
scramble
DRIVE-pPCR, relative recovery
VRK2
HIF1a
KCNJ4
EEF1A2
H19
RPL13A
GAIN
LOSS
CTL
K
5' ETS
28 S
Scramble
Top1 siRNA #1
DRIP-qPCR rel. recovery
ns
RNase A
RNase H
L
Scramble
Top1 siRNA # 2
DRIP-qPCR (% input)
5'ETS
28 S

Figure S2

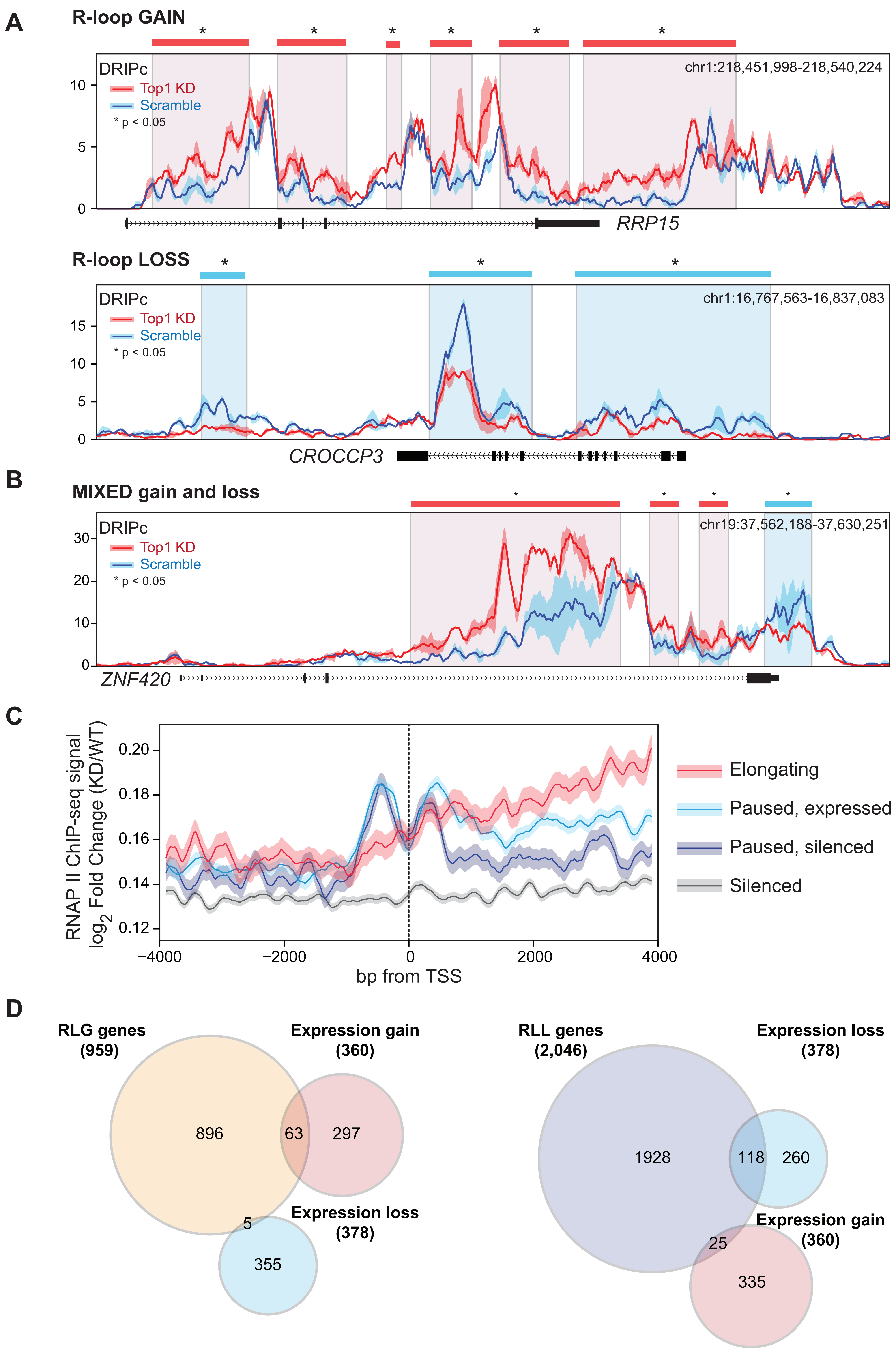

A
R-loop GAIN
DRIPc
Top1 KD
Scramble
* p < 0.05
chr1:218,451,998-218,540,224
RRP15
R-loop LOSS
DRIPc
Top1 KD
Scramble
* p < 0.05
chr1:16,767,563-16,837,083
CROCCP3
B
MIXED gain and loss
DRIPc
Top1 KD
Scramble
* p < 0.05
chr19:37,562,188-37,630,251
ZNF420
C
RNAP II ChIP-seq signal
log2 Fold Change (KD/WT)
bp from TSS
Elongating
Paused, expressed
Paused, silenced
Silenced
D
RLG genes
(959)
Expression gain
(360)
896
63
297
5
Expression loss
(378)
355
RLL genes
(2,046)
Expression loss
(378)
1928
118
260
Expression gain
(360)
25
335

Figure S3

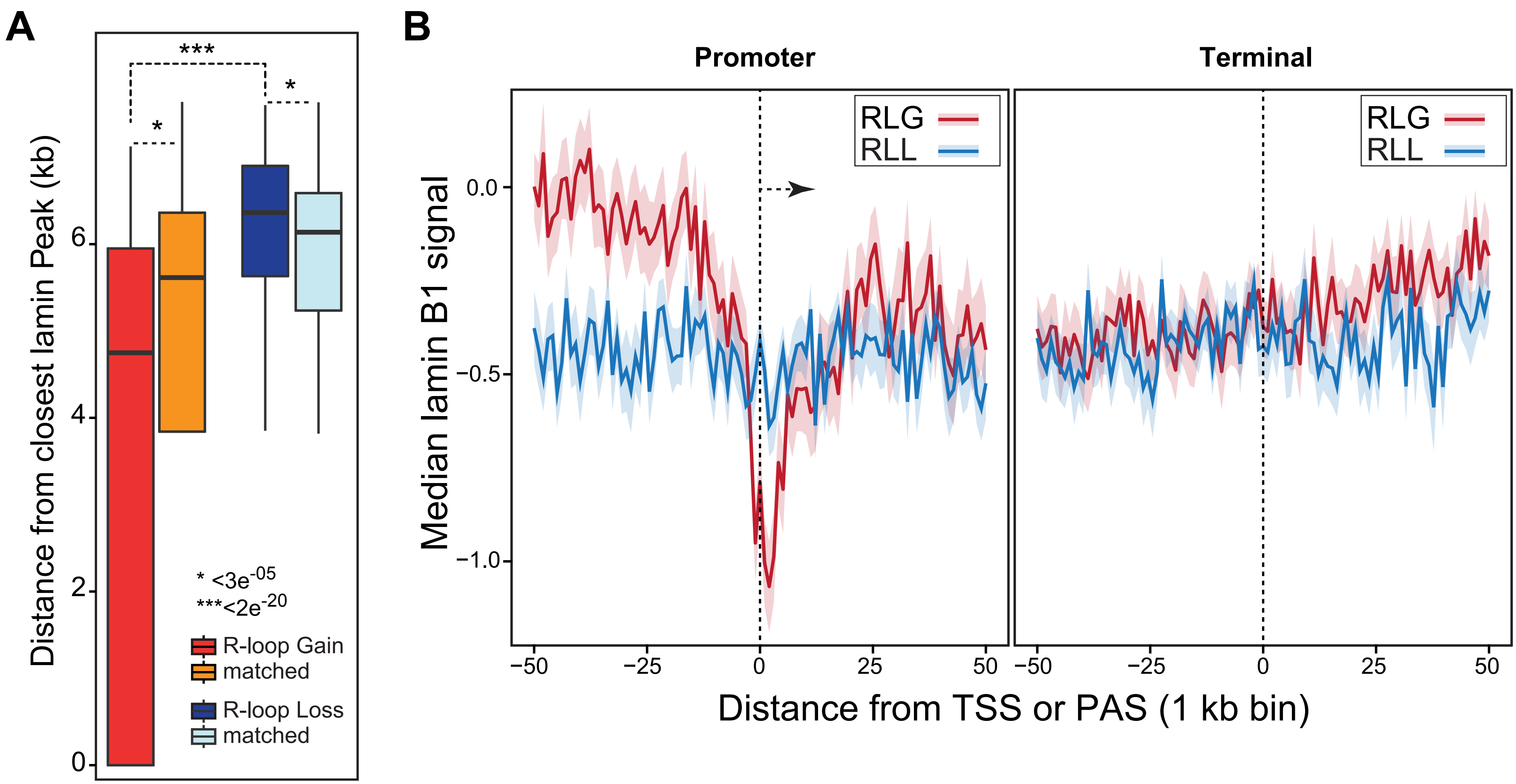

A
***
*
*
Distance from closest lamin Peak (kb)
0
2
4
6
* <3e-05
***<2e-20
R-loop Gain
matched
R-loop Loss
matched
B
Promoter
Terminal
RLG
RLL
Median lamin B1 signal
0.0
−0.5
−1.0
−50
−25
0
25
50
Distance from TSS or PAS (1 kb bin)

Figure S4

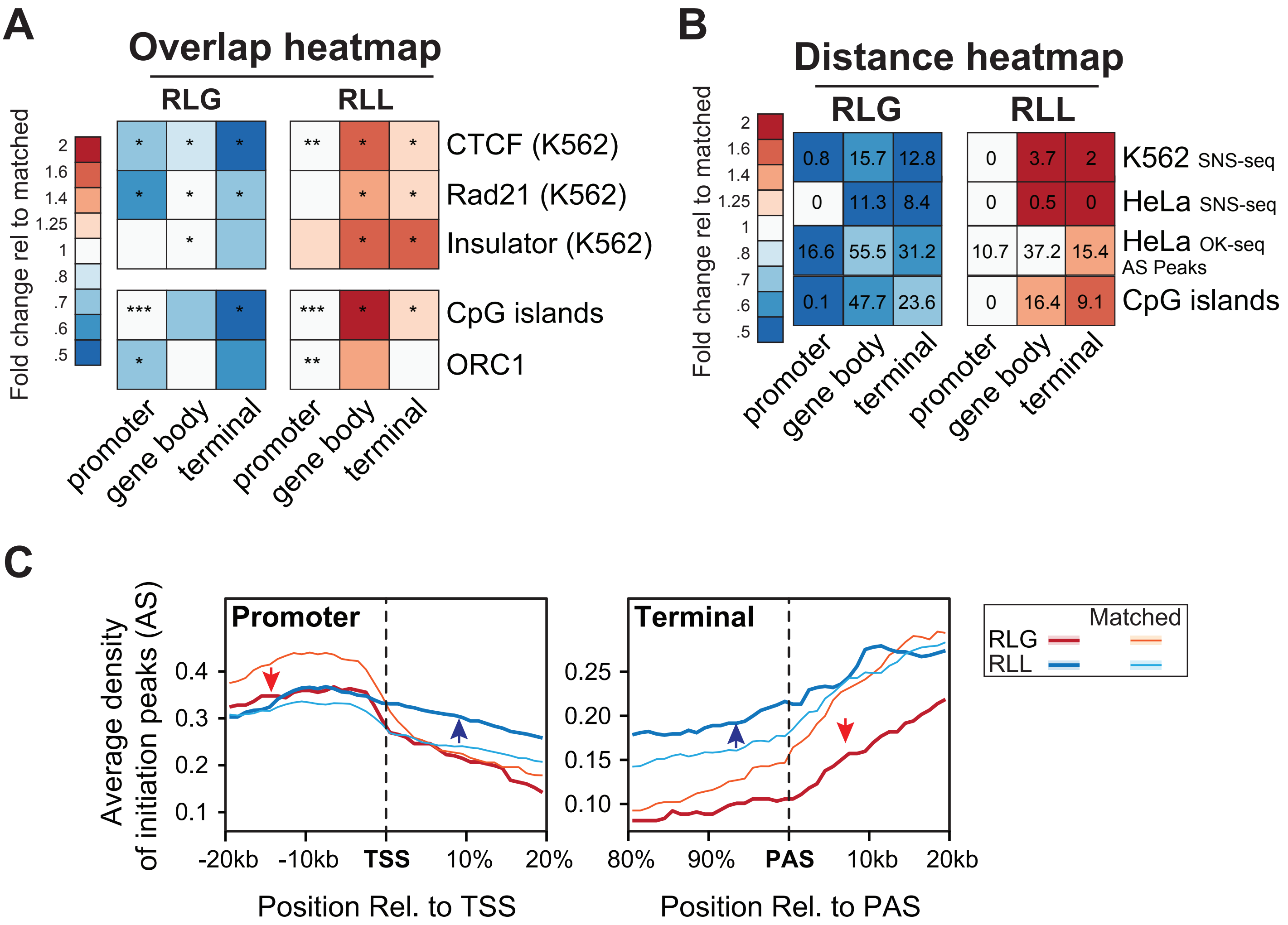

A
Overlap heatmap
RLG
RLL
Fold change rel to matched
2
1.6
1.4
1.25
1
.8
.7
.6
.5
CTCF (K562)
Rad21 (K562)
Insulator (K562)
CpG islands
ORC1
promoter
gene body
terminal
B
Distance heatmap
K562 SNS-seq
HeLa SNS-seq
HeLa OK-seq AS Peaks
CpG islands
0.8 15.7 12.8
0 11.3 8.4
16.6 55.5 31.2
0.1 47.7 23.6
0 3.7 2
0 0.5 0
10.7 37.2 15.4
0 16.4 9.1
C
Promoter
Terminal
Average density of initiation peaks (AS)
-20kb -10kb TSS 10% 20%
80% 90% PAS 10kb 20kb
Position Rel. to TSS
Position Rel. to PAS
Matched
RLG
RLL

Supplementary figure legends

Figure S1: **A**. Quantitation of R loop signal from dot blot shown in Figure 1. Error bars are SE of three independent experiments. A representative DNA loading control is shown at right after agarose gel electrophoresis. **B**. Dot blot and relative quantitation to measure R loop loads in genomic DNA from control or Top1 depleted cells, with and without RNase H pre-treatment. **C**. Dot blot depicting change of global R-loop signal 4, 5, and 6 days after initial Top1 depletion. Samples are indicated at left. **D**. Distribution of DRIPc signal (right) across genomic compartments compared to expected distribution in the genome (left). Color codes are as described in Figure 1C. **E**. Genome browser screenshot illustrating signal “spreading” in the Top1-depleted samples. The R-loop signal for the (+) strand is shown in red. The high-sensitivity peak calls developed to capture signal spreading are found below each track. **F**. XY plot of average log(10) count-normalized DRIPc-seq signals for control samples (scramble – x axis) and top1-depleted samples (y-axis). **G.** Validation by DRIP-qPCR of R-loop gain and loss loci identified by DRIPc-seq. Error bars are SE of three independent experiments. **H.** Validation by DRIP-qPCR R-loop gain (left) and loss (right) upon depletion of Top1 by a second, independent siRNA. The inset above shows a Western blot verifying Top1 depletion. **I.** Validation by DRIP-qPCR of RLL and RLG loci identified by DRIPc-seq 5 and 6 days after Top1 depletion. Error bars represent SE of 2 independent experiments. **J**. Validation of R-loop loss and gain loci identified by DRIPc-seq using S9.6-independent DRIVE-pPCR method. The average and standard deviation of two independent replicates is shown. The *RPL13A* locus represents an invariant control. **K**. DRIP-qPCR analysis of R-loop formation over the 5’ ETS and 28S rDNA regions. Results are average of 3 independent experiments shown with standard deviation. RNase A and RNase H pre-treatments are indicated below. **L.** DRIP-qPCR analysis of R-loop formation over the 5’ ETS and 28S rDNA regions with a second siRNA against Top1. Results are average of 2 independent experiments shown with SEM.

Figure S2: Examples of DRIPc profiles for control and Top1-depleted cells for genes showing gain and loss (**A**) or mixed R-loop changes (**B**) after Top1 knockdown. Stars indicate statistically significant differences. **C**. Ratio plots of the RNA polymerase II ChIP-seq signal between Top1-depleted and control cells around the TSS of specific gene categories according to expression and pausing status. Top1 depletion causes an increase in RNAPII levels around the TSS of paused genes. Peak shape differences between our and previous studies [30] are likely due to the use of different antibodies (we used a pan-RNAPII Ab whereas others used an anti-S5P RNAPII Ab). This allowed us to observe progressive RNAPII accumulation downstream of the TSS, consistent with RNAPII encountering difficulty during elongation in the absence of Top1. **D**. Venn diagrams depicting the overlap between RLG and RLL genes and genes undergoing up or down regulation in Top1-depleted cells. Differentially expressed genes were identified with a 1.5-fold up or down minimal threshold (and adjusted p-value<0.05).

Figure S3: A. Distance between the top 100 RLG and RLL peaks (as measured to signal gains and losses) and LADs compared to matched controls. Statistical significance was measured by Wilcoxon test. **B**. Metaplots of Lamin B1 signal over the promoters and terminals of RLG and RLL genes measured over a +/- 50 kb window. Genes are aligned at their TSS or polyadenylation sites (PAS). Values are median and shown with standard deviation (shaded).

Figure S4: **A.** Heatmap of enrichment or depletion of RLG and RLL peaks over specific chromatin features as measured by peak overlap (CTCF, Rad21, Insulator, CpG Islands, and ORC1). Color codes and description is as Figure 4A. Stars indicate the extent of overlap between R-loop peaks and each chromatin feature (* 10-25%; ** 25-50%; *** >50%; no star <10%). All values are significant with p-value < 0.08 (Monte-Carlo) **B.** Heatmap of distance of RLG and RLL peaks from OK-seq, SNS-seq, and CpG island peaks. The numbers indicate the

median distance (in kb) from RLG and RLL peaks and each feature. **C**. Average density of OK-seq replication initiation zones (AS) were plotted around promoter and terminal regions for RLL, RLG and corresponding matched invariant genes. Red arrows indicate loss of origin density for RLG genes compared to matched genes. Blue arrows indicate gain of origin densities compared to matched genes.
