# Supplementary material for: DNA Topoisomerase I differentially modulates R-loops across the human genome
Source: Genome Biol. 2018 Jul 30;19:100. doi: 10.1186/s13059-018-1478-1 (PMC6066927; doi:10.1186/s13059-018-1478-1)
Supplement: Supplementary file 2 — Figure S5. Validation of cell cycle block using an independent Top1 siRNA. A. Representative images of ki-67 staining for Top1-depleted and control cells. Cells were counter-stained with DAPI. B. Quantification of ki-67 staining (350 cells for each sample from one representative experiment). C. Cell cycle analysis for Top1-depleted cells and controls. Results are from one representative experiment. D. Cytofluorimetric profile for control and Top1-depleted cells. X-axis is propidium iodide signal, y-axis is cell count. Figure S6. (A) R-loop levels measured by DRIP-qPCR at nine different loci across the G2/M, G1 and S phases of the cell cycle are showed here normalized to R-loop levels in asynchronous cells. Results are shown as average and standard deviation from two experiments. (B) Re-analysis of R-loop mapping data from MCF7 cells before and 2 and 24 h after stimulation with estradiol (data from GSE81851). Top: overlap between R-loop peaks and RLG and RLL genes. Bottom: Total length covered by R-loop peaks in RLG and RLL peaks. (C and D) Analysis of replication timing at a range of Mixed/no change loci (C) and RLG loci (D) in Top1-depleted cells and controls. Recovery of BrdU-labeled immuno-precipitated DNA for each phase was normalized based on total signal for each sample. Error bars are SE of two replicates. Red and grey shading indicate genes with significant and non-significant replication timing delays, respectively. Table S1. Number of R-loop peaks and genes after peak calling with the standard method [4] or the high sensitivity method implemented here. The numbers are broken down between loci that undergo R-loop gains, Rloop losses, no change, or both. is propidium iodide signal, y-axis is cell count. (PDF 17536 kb) [file 13059_2018_1478_MOESM2_ESM.pdf]

Figure S5

A

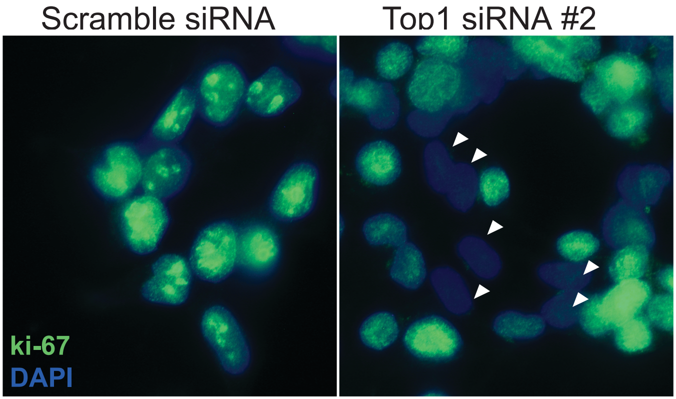

B

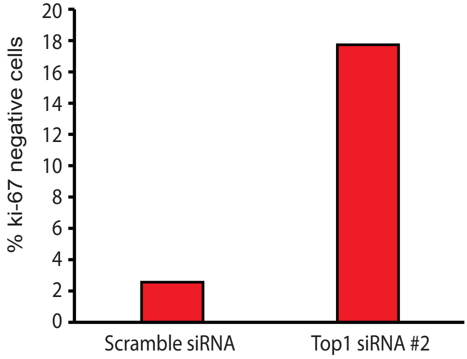

C

PHASE	Scramble siRNA	Top1 siRNA #2
G0/G1	41.8%	67.6%
S	24.1%	12.2%
G2/M	33.3%	19.6%
APOPT.	0.8%	0.6%

D

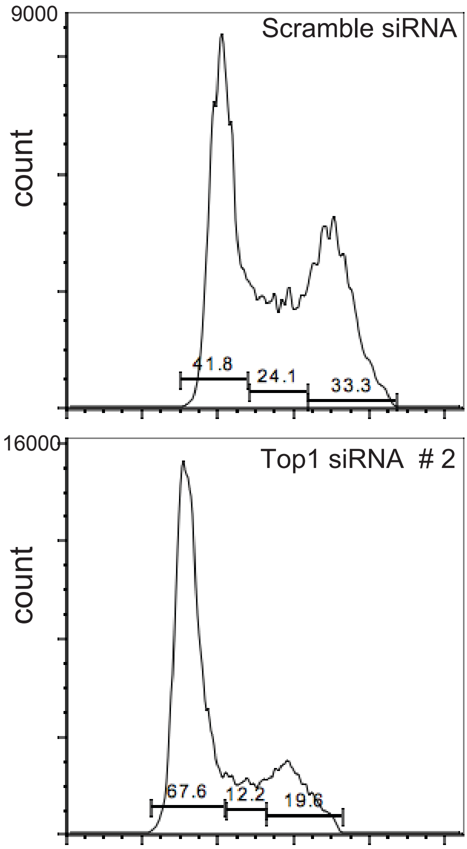

Figure S6

A

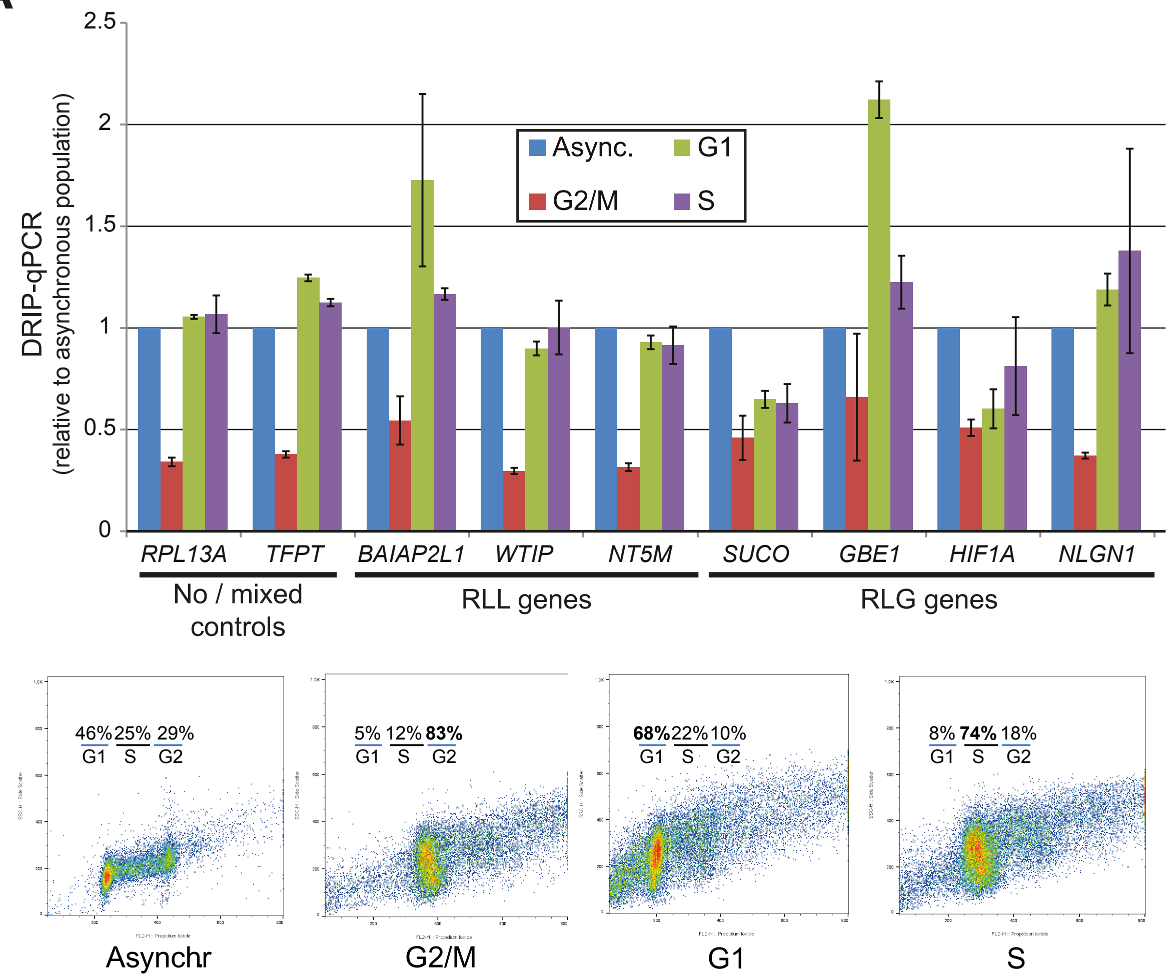

B

Gene Type	Total Genes	Percent Intersect		
		0 hr	2 hr	24 hr
RLG	959	40%	53%	47%
RLL	2,046	48%	55%	52%

Gene Type	Total Gene Length (Mbp)	Total Intersect Length (Mbp)		
		0 hr	2 hr	24 hr
RLG	170.54	1.45	2.88	2.10
RLL	135.78	7.88	10.52	9.46

C

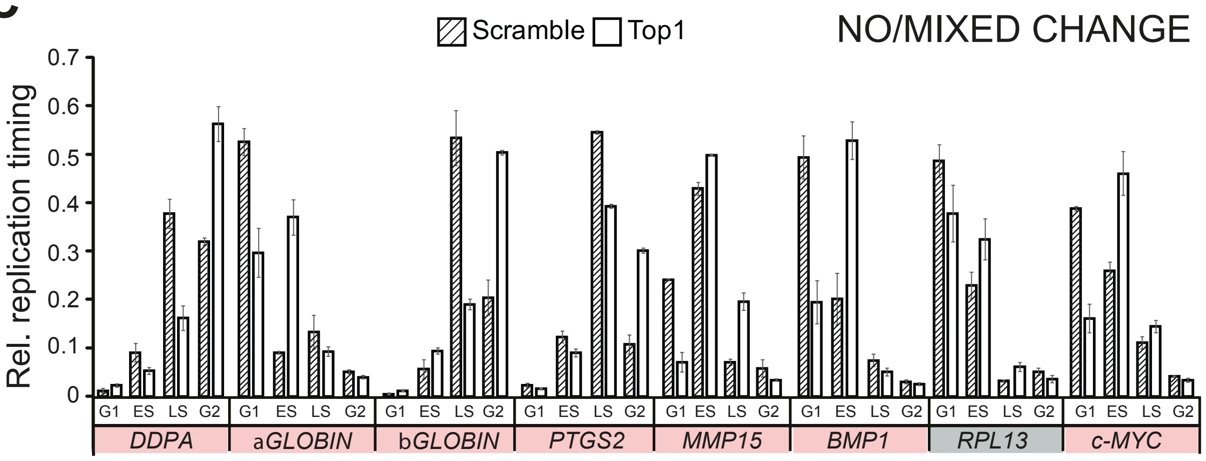

D

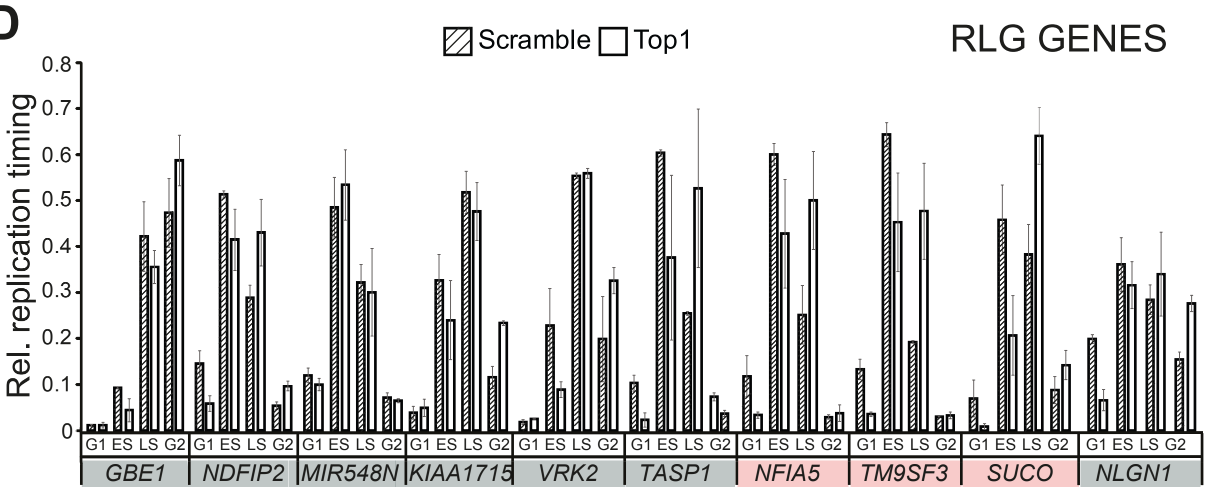

Table S1

R-loop gain: Fold change of >= 1.25 and adjusted p-value <= 0.1
R-loop loss: Fold change of <= 0.80 and adjusted p-value <= 0.1

	R-loop PEAKS	
	Standard	Higher Sensitivity
GAINS	1,722	15,112
NO CHANGE	66,257	371,864
LOSS	1,087	12,977
TOTAL	69,066	399,953
% feature affected by Top1 KD	4.07%	7.02%

	GENES (total 25,556)	
	Standard	Higher Sensitivity
GAINS (RLG)	1,002	959
NO / MIXED CHANGE	12,161	11,839
LOSS (RLL)	613	2,046
TOTAL	13,776	14,844
% feature affected by Top1 KD	6.32%	11.76%

Supplementary figure legends

Figure S5: Validation of cell cycle block using an independent Top1 siRNA. **A**. Representative images of ki-67 staining for Top1-depleted and control cells. Cells were counter-stained with DAPI. **B**. Quantification of ki-67 staining (350 cells for each sample from one representative experiment). **C**. Cell cycle analysis for Top1-depleted cells and controls. Results are from one representative experiment. **D**. Cytofluorimetric profile for control and Top1-depleted cells. X-axis is propidium iodide signal, y-axis is cell count.

Figure S6: (**A**) R-loop levels measured by DRIP-qPCR at nine different loci across the G2/M, G1 and S phases of the cell cycle are showed here normalized to R-loop levels in asynchronous cells. Results are shown as average and standard deviation from two experiments. (**B**) Re-analysis of R-loop mapping data from MCF7 cells before and 2 and 24 hours after stimulation with estradiol (data from GSE81851). Top: overlap between R-loop peaks and RLG and RLL genes. Bottom: Total length covered by R-loop peaks in RLG and RLL peaks. (**C and D**) Analysis of replication timing at a range of Mixed/no change loci (**C**) and RLG loci (**D**) in Top1-depleted cells and controls. Recovery of BrdU-labeled immuno-precipitated DNA for each phase was normalized based on total signal for each sample. Error bars are SE of two replicates. Red and grey shading indicate genes with significant and non-significant replication timing delays, respectively.

Supplementary table legends

Table S1: Number of R-loop peaks and genes after peak calling with the standard method (Sanz et al., 2016) or the high sensitivity method implemented here. The numbers are broken down between loci that undergo R-loop gains, R-loop losses, no change, or both. is propidium iodide signal, y-axis is cell count.
