# Supplementary material for: DNA Topoisomerase I differentially modulates R-loops across the human genome
Source: Genome Biol. 2018 Jul 30;19:100. doi: 10.1186/s13059-018-1478-1 (PMC6066927; doi:10.1186/s13059-018-1478-1)
Supplement: Supplementary file 3 — Review history. (DOCX 320 kb) [file 13059_2018_1478_MOESM3_ESM.docx]

First round of Review:

**Reviewer 1:**

**Are the methods appropriate to the aims of the study, are they well described, and are necessary controls included?**

Yes, except where noted below.

**Are the conclusions adequately supported by the data shown?**

Yes, except where noted below.

**Are sufficient details provided to allow replication and comparison with related analyses that may have been performed?**

Yes

**Does the work represent a significant advance over previously published studies? Please explain.**

I believe the advance is significant. While the role of topoisomerases in regulation of R-loops has been addressed previously in several systems, this study performs an in depth analysis of genomic features affected by Top1 loss and reaches several new conclusions.

**Is the paper of broad interest to others in the field, or of outstanding interest to a broad audience of biologists?**

Yes: Of interest to biologists interested in epigenetics and R-loops

**Comments to author:**

Manzo et al examine the global effects of topoisomerase I (Top1) loss on the accumulation of R-loops. Upon RNAi of Top1, the authors find regions of R-loop loss and larger regions of R-loop gain. Gains and losses occur in distinct genomic regions dependent on gene structure and length, and differentially correlate with epigenetic marks. The authors go into considerable depth to characterize the genomic features that coincide with each region and the genomics analyses appear to be well done and thorough. With one caveat noted below, the data and analyses support the authors' conclusions and provide interesting insights into R-loops in human cells.

Major Point

1. The authors note that a significant fraction of cells enter into a quiescent (G0) state upon Top1 RNAi (Figure 6D and E), which raises the possibility that increases and decreases in R-loops at various loci throughout the genome may be partially due to an indirect effect of cell cycle exit. To address this issue, the authors synchronized cells using an arrest/release experiment and tested R-loop accumulation in G1, S, and G2/M, observing no major effect on R-loop accumulation at RLG or RLL regions, outside of a depletion at M phase, where transcription is minimal. However, this experiment does not mimic the quiescent state. To test for effects of quiescence on R-loop gain or loss, the authors should repeat this experiment in G0 cells (using serum starvation) with and without Top1 knockdown.

Minor Points

1. The authors should avoid claiming Top1 is depleted to "undetectable levels" when a weak band is visible in their Top1 RNAi western blot in Figure 1.
2. The dot blot in Figure 1B needs a loading control to allow valid interpretation of the data.
3. RLG peaks were enriched for elongation (K36me3, K79me2) and heterochromatin (K9me3) marks. Presumably these are mostly non-overlapping genomic regions. However, on page 8 lines 52-56, the authors write "These observations are consistent with RLG genes being long and highly expressed and occupying gene-poor neighborhoods (Fig. 2) that are more likely to associate with heterochromatic regions." Very few highly expressed genes are found within heterochromatic regions. The authors should clarify this point.
4. On page 11, the authors write "Analysis of replication timing data (Repli-seq) confirmed that RLL peaks replicate early, mostly in G1 and S1 phases (Fig. 5F)." I do not understand this comment - there is no DNA replication in G1, by definition. Presumably this refers to nomenclature from a previous paper, but no explanation is provided.
5. The authors need to include data demonstrating effective synchronization of the cell cycle (currently listed as "data not shown") to allow proper interpretation of the synchronization experiments.
6. siRNA sequences should be included.

**Reviewer 2**

**Are the methods appropriate to the aims of the study, are they well described, and are necessary controls included?**

Mostly they are included with a few exceptions/suggestions: e.g. the dot blot in Figure 1B: What is the loading control for the S9.6 signal? How can we be sure of equal loading of genomic DNA? The results suggest that the overall effect is an elevation of R-loops. Does the proportion of RLG exceed RLL, or is it cell cycle dependent?

**Are the conclusions adequately supported by the data shown?**

Yes

**Are sufficient details provided to allow replication and comparison with related analyses that may have been performed?**

Yes

**Does the work represent a significant advance over previously published studies? Please explain.**

It is well established that TOP1 regulates R-loop homeostasis but little is known about how this dynamic regulation is achieved at a global level. In contrast to many other studies, the authors challenged HEK293 cells with an acute and transient TOP1 depletion coupled with mapping R-loops genome-wide using S9.6 based DRIP. Interestingly, transient TOP1 depletion led to both an increase and a decrease in R-loop levels, based on their genome-wide distribution and neighbourhood. The authors identify two gene classes: R-loop gains for long, highly transcribed, genes located in gene-poor regions anchored to Lamin B1 domains and close to H3K9me3 patches. Another class of R-loop losses for gene-rich regions overlapping H3K27me3 marked active replication initiation regions. The authors consequently propose a role for TOP1 in regulating origin firing. Overall, this is a beautiful study illustrating context-dependent R-loop regulation by TOP1 and will open many avenues for further follow-up work. There are a few points that need to be addressed to improve the manuscript, as detailed in my letter to the authors below

**Is the paper of broad interest to others in the field, or of outstanding interest to a broad audience of biologists?**

Yes, it challenges the common view that TOP1 depletion simply leads to an increase in R-loops.

**Comments to Author**

It is well established that TOP1 regulates R-loop homeostasis but little is known about how this dynamic regulation is achieved at a global level. In contrast to many other studies, the authors challenged HEK293 cells with an acute and transient TOP1 depletion coupled with mapping R-loops genome-wide using S9.6 based DRIP. Interestingly, transient TOP1 depletion led to both an increase and a decrease in R-loop levels, based on their genome-wide distribution and neighbourhood. The authors identify two gene classes: R-loop gains for long, highly transcribed, genes located in gene-poor regions anchored to Lamin B1 domains and close to H3K9me3 patches. Another class of R-loop losses for gene-rich regions overlapping H3K27me3 marked active replication initiation regions. The authors consequently propose a role for TOP1 in regulating origin firing. Overall, this is a beautiful study illustrating context-dependent R-loop regulation by TOP1 and will open many avenues for further follow-up work. There are a few points that need to be addressed to improve the manuscript:

1. The results describe R-loop profiling following acute short-term 24 hr depletion of TOP1. How is this compared to a more prolonged deprivation of TOP1? Will the RLG and RLL profile be maintained or will it change to a different profile? In case it changes, which approach is more physiologically relevant?

2. A concern with stable TOP1 knockdown is cellular adaptation. The authors may therefore consider a time course experiment. Will the profile of RLG and RLL be the same following an additional 24 hr or 48 hr depletion of TOP1?

3. There is some concern in the field from relying only on the S9.6 antibodies for R-loop profiling. Is it possible to validate, at least, the key results using other methods (e.g. RNA/DNA binding domain of RNaseH1)?

4. The dot blot in Figure 1B: What is the loading control for the S9.6 signal? How can we be sure of equal loading of genomic DNA? The results suggest that the overall effect is an elevation of R-loops. Does the proportion of RLG exceed RLL, or is it cell cycle dependent?

5. Mitochondrial DNA replication timing was not affected by TOP1 knockdown. It is not clear which TOP1 isoform the siRNA targets, is it the nuclear or the mitochondrial isoform, or both? A Western blotting for both isoforms would be useful to include.

**Reviewer 3**

**Are the methods appropriate to the aims of the study, are they well described, and are necessary controls included?**

Yes

**Are the conclusions adequately supported by the data shown?**

no, see comments below

**Are sufficient details provided to allow replication and comparison with related analyses that may have been performed?**

no, see comments below.

**Does the work represent a significant advance over previously published studies? Please explain.**

Potential but preliminary

**Is the paper of broad interest to others in the field, or of outstanding interest to a broad audience of biologists?**

No

**Comments to Author**

Manzo et al. use DRIPc-seq to compare the landscape of R-loops between wild type human cells and cells depleted of Topoisomerase 1 (Top1). They identified two classes of genes that were affected by Top1 depletion, ones that either gain or lose R-loop signal. They observe a correlation of between the regions that gain R-loops with highly transcribed long genes in gene-poor regions, anchored to Laminin B1 domains. In contrast, R-loops depleted regions in Top1 depleted cells correlated with gene-rich regions, rich in replication-initiations regions.

The manuscript is written for a sequencing-savvy readership, and as result is quite hard to understand (particularly text describing figures 4-5). This lack of clarity makes the significance of the correlations unclear, consequently also weakening the strength of the conclusions. A more detailed explanation of the experimental design and a better presentation of the results and conclusions in the result section will greatly improve the paper. In general, stating the main conclusion for each paragraph in the results will greatly help the reader understand the manuscript.

Additional revisions.

- Indicate hybrid signal or s9.6 signal in the figure 1b.

- Change color in S1C and 1c since terminators and promoters are barely distinguishable

- The description of the DRIP-seq results in WT vs Top1 depletion is confusing. My understanding is that WT generates 70.000 peaks of hybrids covering around 200MB of genome. Then the authors describe data analysis on the Top1 depletion, concluding that classical peak calling detects differences from WT in just a very small percentages of regions (4%). Analysis of single peaks suggested that the signal in Top1 depleted cells spread from regions in WT. However, these data are not shown in a figure nor is it stated clearly?

- Then a more sensitive peak calling software (which presumably was used in both WT and Top1 samples?) revealed that gain and loss occurred at 28000 peaks (relative to WT?). It is not clear how those 28000 peaks are just 7% of the total peaks (70000). Also, R-loops in WT cells cover 200Mb. In the top1 depleted cells regions of R-loop gain affected 100MB and loss 36MB (from figure 1C). How are those numbers consistent with Top1 depletion only affecting a small subset of hybrid regions?

- The authors jump from signal spreading (which presumably means peaks covering more DNA sequence), to increase/decrease of in the region, which is very confusing. Does Top1 depletion make longer hybrid regions, or increase the number of hybrids in a region? These points must be described and discussed more clearly.

- The rationale and intro for figure 1E (rDNA analysis) should be presented before the data.

- In figure 4A and 4B and following figures, the authors compare the RLG and RLL with a subset of hybrid controls that match the characteristics of the RLG and RLL regions, but don't show changes in R-loop levels upon Top1 depletion. This subset is different from the one used in the previous figures. It is not clear why the control subset was changed and how the result will look using the previous control subset.

- There are no arrows in figure 4D as indicated in figure legend. It Is not clear what is the difference between the 3 panels on the right and the 3 on the left.

- Figure 5D terminators are called terminal. Is there a specific reason for this new nomenclature?

- The results on the cell cycle delay presented in figure 6 do not appear connected with the regions of gain and loss of R-loops upon Top1 depletion. There is no difference in phenotype between the regions of loss (fig6F) and the control regions used (fig. S6b). How are these results connected with the previous observations? The results in figure 6 do not appear to corroborate or extend the conclusions from the previous figures.

- There are numerous typos in the manuscript that need to be corrected.

**Authors’ response to reviewers**

***Reviewer #1***:

*Manzo et al examine the global effects of topoisomerase I (Top1) loss on the*

*accumulation of R-loops. Upon RNAi of Top1, the authors find regions of R-loop loss and larger*

*regions of R-loop gain. Gains and losses occur in distinct genomic regions dependent on gene*

*structure and length, and differentially correlate with epigenetic marks. The authors go into*

*considerable depth to characterize the genomic features that coincide with each region and the*

*genomics analyses appear to be well done and thorough. With one caveat noted below, the data*

*and analyses support the authors' conclusions and provide interesting insights into R-loops in*

*human cells.*

We thank this reviewer for noting that the study was “well done and thorough” and “provides interesting insights into R-loops in human cells”

*Major Point*

*1. The authors note that a significant fraction of cells enter into a quiescent (G0) state upon*

*Top1 RNAi (Figure 6D and E), which raises the possibility that increases and decreases in Rloops*

*at various loci throughout the genome may be partially due to an indirect effect of cell cycle*

*exit. To address this issue, the authors synchronized cells using an arrest/release experiment and*

*tested R-loop accumulation in G1, S, and G2/M, observing no major effect on R-loop*

*accumulation at RLG or RLL regions, outside of a depletion at M phase, where transcription is*

*minimal. However, this experiment does not mimic the quiescent state. To test for effects of*

*quiescence on R-loop gain or loss, the authors should repeat this experiment in G0 cells (using*

*serum starvation) with and without Top1 knockdown.*

This point is well taken and we gave the proposed experiments a strong effort. Before detailing

these experiments, we will note two things. First, the proportion of ki-67 negative G0 cells as a

result of Top1 depletion was minor – 15% (Figure 6E). Thus it is unlikely that the R-loop losses

and gains that we observed could simply be due to this small population of cells. Second, serumstarved

G0 cells may be in a quiescent state quite distinct from the one elicited by Top1 depletion

which are cultured in 10% serum. In fact, our expectations were that serum-starved cells would

show a global decrease in R-loops because of reduced transcription levels, much like we observed

in G2/M sorted cells.

Nonetheless, we went ahead and attempted to serum starve HEK293 cells by first growing them

for 18 hours in 0.1% serum following conditions published by Bunch et al. (2015) in Nature

Communications. This was followed by flow cytometry to determine if a significant fraction of

the cell population was now accumulating in G0/G1. No significant differences were observed

although clear morphological changes (aggregation) were noticed. This was followed by 4

independent attempts to achieve G0/G1 synchronization by serum starvation by changing the time

(from 17 all the way to 62 hours) under which the cells were grown at 0.1% serum and changing

the confluency of the culture before starvation. No significant change in the cell cycle distribution

profile was observed under any condition. To more directly read out any G0 arrest, we also

performed anti-ki-67 immunocytochemistry. No significant increase in the proportion of ki-67-

negative cells could be detected. Thus, despite significant efforts over the past two months, we

concluded that HEK293 cells cannot easily be synchronized in G0 upon serum starvation.

As a way to address the point raised by the referee, we took advantage of a recent dataset by the

Cimprich laboratory in which R-loops were mapped using DRIP-seq in a population of breast

cancer cells (MCF7) in the presence or absence of estrogen (Stork et al., (2016) published in

eLife). Without estrogen, MCF7 cells essentially become quiescent and 85% of the cells

accumulate in G0/G1. Upon stimulation with estrogen, cells undergo a rapid burst of transcription

followed by a resumption of the cell cycle (FACS profiles to support this are shown in Figure 1 –

Figure supplement 2 of the original paper). We reasoned that if the R-loop gains and losses

observed upon Top1 depletion are in fact caused by the G0 arrest, then a similar, perhaps even

amplified, pattern should be observed in MCF7 cells. Specifically, we should observe that RLG

genes are R-loop positive in unstimulated MCF7 cells and become less R-loop positive upon reentry

in the cell cycle. Vice-versa, we should observe that RLL genes are R-loop negative in

unstimulated MCF7 cells but become R-loop positive upon estrogen stimulation.

To test this, we obtained the raw DRIP-seq data, mapped it and called peaks against input signal

using identical thresholds and methods as those published by Stork et al., (2016). The samples

included unstimulated MCF7 cells and MCF7 cells 2 and 24 hours post-stimulation with

estradiol. We then determined the overlap between R-loop peaks in each sample and both RLG

genes (n=959) and RLL genes (n=2,046). As can be seen in Table 1 below, the proportion of

RLG and RLL genes that intersect with R-loops (i.e. at least on R-loop peak in the gene) is

relatively constant through time. Thus we do not observe that the proportion of R-loop-positive

RLG genes decreases with re-entry into the cell cycle as would be expected if R-loop formation at

RLG genes was a property of the G0/G1 state. Vice-versa, we do not observe a significant

increase in the proportion of R-loop positive RLL genes, as would be expected if R-loop

formation was only allowed outside the G0/G1 phase in RLL genes.

Table 1. Percent intersect between RLG or RLL genes and MCF7 DRIP peaks from Stork et al., (2016).

Similar results were obtained when we measured the total R-loop load (i.e. length occupied by Rloop

peaks) of RLL and RLG genes. Table 2 shows the data.

Table 2. Length intersect between RLG or RLL genes and MCF7 DRIP peaks from Stork et al., (2016).

Overall, we conclude that the patterns of R-loop losses and gains observed upon Top1 depletion are unlikely to simply reflect shifts in cell cycle patterns and are more likely to result from the response to Top1 depletion.

We have introduced a slightly abbreviated version of our analysis of Stork et al. (2016) in the manuscript (page 13) and included the two tables above in Figure S6.

*Minor Points*

*1. The authors should avoid claiming Top1 is depleted to "undetectable levels" when a weak band is visible in their Top1 RNAi western blot in Figure 1.*

We removed this mention and clarified the experimental protocol we followed. The first sentence of the Results section now states “we used siRNA transfection to efficiently silence Top1 in human HEK293 cells for five days”.

*2. The dot blot in Figure 1B needs a loading control to allow valid interpretation of the data.*

We thank the reviewer for pointing this out. To ensure equal loading, we systematically withdrew an aliquot of DNA prior to application on the membrane and loaded it on an agarose gel. Densitometry was used to confirm that all samples were equally digested and of equal intensities. A statement about loading control was added to the “Dot Blot Analysis” paragraph of the Methods section. A representative example is now included in Figure S1A.

*3. RLG peaks were enriched for elongation (K36me3, K79me2) and heterochromatin (K9me3) marks. Presumably these are mostly non-overlapping genomic regions. However, on page 8 lines 52-56, the authors write "These observations are consistent with RLG genes being long and highly expressed and occupying gene-poor neighborhoods (Fig. 2) that are more likely to associate with heterochromatic regions." Very few highly expressed genes are found within heterochromatic regions. The authors should clarify this point.*

Perhaps the confusion here comes from the use of the word “heterochromatic” which was not meant to indicate an association to constitutive centromeric heterochromatin. We modified the sentence to: “These observations are consistent with RLG genes being long and highly expressed and occupying gene-poor neighborhoods (Fig. 2) that are more likely to include heterochromatinlike features." We thank the reviewer for allowing us to clarify this point.

*4. On page 11, the authors write "Analysis of replication timing data (Repli-seq) confirmed that RLL peaks replicate early, mostly in G1 and S1 phases (Fig. 5F)." I do not understand this comment - there is no DNA replication in G1, by definition. Presumably this refers to nomenclature from a previous paper, but no explanation is provided.*

The reviewer is correct that the reference to G1 is confusing and is taken from the original Repliseq paper, which showed that BrdU incorporation begins at the earliest in late G1 (G1b) at certain loci. Whether or not this late G1 incorporation truly represents replication in G1 or in very early S is a matter of debate and beyond our point here. We clarified this statement by citing the original publication (now Ref 41) and modifying the sentence to state that “Analysis of replication timing data (Repli-seq, [41]) confirmed that RLL peaks replicate as early as late G1 (G1b) and S1 phases (Fig. 5F)." In the next sentence, a mention of replication “in G1” was changed to replication in “late G1”. Figure 5 was modified to include “G1b” instead of “G1”

*5. The authors need to include data demonstrating effective synchronization of the cell cycle (currently listed as "data not shown") to allow proper interpretation of the synchronization experiments.*

As requested, representative FACS screenshots are now included as part of Figure S6A.

*6. siRNA sequences should be included.*

We now include the specific siRNA codes from the vendor ThermoFisher.

***Reviewer #2***:

*It is well established that TOP1 regulates R-loop homeostasis but little is known about how this dynamic regulation is achieved at a global level. In contrast to many other studies, the authors challenged HEK293 cells with an acute and transient TOP1 depletion coupled with mapping R-loops genome-wide using S9.6 based DRIP. Interestingly, transient TOP1 depletion led to both an increase and a decrease in R-loop levels, based on their genome-wide distribution and neighbourhood. The authors identify two gene classes: R-loop gains for long, highly transcribed, genes located in gene-poor regions anchored to Lamin B1 domains and close to H3K9me3 patches. Another class of R-loop losses for gene-rich regions overlapping H3K27me3 marked active replication initiation regions. The authors consequently propose a role for TOP1 in regulating origin firing. Overall, this is a beautiful study illustrating context-dependent R-loop regulation by TOP1 and will open many avenues for further follow-up work. There are a few points that need to be addressed to improve the manuscript.*

We appreciate the reviewer’s positive comments and in particular his/her recognition of a context-dependent regulation of R-loops by Top1 which has so far never been described for this enzyme or any other.

*1. The results describe R-loop profiling following acute short-term 24 hr depletion of TOP1. How is this compared to a more prolonged deprivation of TOP1? Will the RLG and RLL profile be maintained or will it change to a different profile? In case it changes, which approach is more physiologically relevant?*

*2. A concern with stable TOP1 knockdown is cellular adaptation. The authors may therefore consider a time course experiment. Will the profile of RLG and RLL be the same following an additional 24 hr or 48 hr depletion of TOP1?*

Here we address both points 1 and 2 together. First as a point of clarification, all of our R-loop mapping experiments were performed 5 days post Top1 depletion, not 24 hours. As was stated in the Methods section, we performed an initial siRNA transfection and “48 hours after the first transfection, one fifth of the cells were transfected again in a similar manner. Cells were harvested 72 hours after the second transfection for all subsequent analysis”. To clarify this point further, we added that “R-loop increase was detectable 5 and 6 days post Top1 depletion but could not yet be detected 4 days post-transfection (Supplemental Figure S1C). To capture the early R-loop response to Top1 depletion all subsequent experiments were performed 5 days after initial Top1 depletion.” in the first paragraph of the Results section. Nonetheless, the reviewer is correct that the kinetics of the Top1 effect is of interest and we now present new data addressing this point. In all cases, we performed an initial siRNA transfection and followed up with a second transfection after 48 hours. The samples were then harvested 2, 3, or 4 days after this second transfection. On a global level (using S9.6 dot blots as a readout), a significant increase in R-loop loads is detectable 5 and 6 days after initial transfection but not after 4 days. Likewise, no change was detected after just two days post initial transfection (not shown). Using DRIP-qPCR at various RLG and RLL loci, we confirm that the trends towards gains and losses are visible 5 and 6 days post depletion. Thus it appears that the phenomenon of R-loop gains and losses requires about 4 days to build. This information was added to the first paragraph of the Results section and in Figure S1C and S1I.

*3. There is some concern in the field from relying only on the S9.6 antibodies for R-loop profiling. Is it possible to validate, at least, the key results using other methods (e.g. RNA/DNA binding domain of RNaseH1)?*

With over a decade of experience in R-loop mapping using a variety of techniques, we should first make clear that we fully stand behind the strength of the S9.6 antibody. When properly used, the S9.6 antibody represents an extremely powerful reagent to analyze R-loops on a global scale. Nonetheless, we agree that independent methods are always welcome. The Chedin lab has embarked on a comprehensive R-loop mapping effort using non-denaturing bisulfite treatment followed by single molecule sequencing in an S9.6-independent manner. The results of the study are currently being written for publication. On the technical side, one of the key conclusions is that aggregate single-molecule R-loop footprinting data recapitulates DRIPc-seq data to an extraordinary extent. Thus we feel very strongly that the maps shown here are accurate. With this being said, we followed the reviewer’s suggestion and used the catalytically-inactive human RNASEH1 enzyme to capture R-loops and quantify them using qPCR. This follows the DRIVE method initially developed by the Chedin laboratory (Ref 5). For this experiment, we targeted three loci showing R-loop loss and two loci showing R-loop gains plus an invariant control. As can now be seen in Fig S1J, the global trends towards gain and loss were recapitulated. The magnitude of the effects were slightly lower than those measured by DRIP which is consistent with our experience showing that the RNASEH1-mediated capture is significantly less sensitive than S9.6-based pulldowns.

*4. The dot blot in Figure 1B: What is the loading control for the S9.6 signal? How can we be sure of equal loading of genomic DNA? The results suggest that the overall effect is an elevation of R-loops. Does the proportion of RLG exceed RLL, or is it cell cycle dependent?*

The reviewer’s concern about loading control is well taken and was addressed in response to Reviewer 1 (minor point #2). The reviewer correctly states that the overall effect of Top1 depletion is a net R-loop gain genome-wide, as revealed by dot-blots and by our DRIPc-seq mapping. These points are made in Figure1 (B-E) and Figure S1 (A-D). We have not investigated the cell cycle dependency in detail at a global level. However, our findings on a subset of loci undergoing gains or losses did not reveal strong global trends related to cell cycle (Figure S6A), other than reduced R-loops in G2/M, as expected from the corresponding lower transcriptional output.

5. Mitochondrial DNA replication timing was not affected by TOP1 knockdown. It is not clear which TOP1 isoform the siRNA targets, is it the nuclear or the mitochondrial isoform, or both? A Western blotting for both isoforms would be useful to include.

The reviewer raises a good point. The siRNAs were specific for the nuclear Top1 gene (TOP1) which we now specifically state in the Methods section. The total RNA-seq data accompanying the manuscript confirms strong nuclear Top1 down-regulation and shows that the expression of the mitochondrial Top1 gene, TOP1MT, was unaffected. We added this information in the Methods section. A screenshot illustrating this is shown here.

Total RNA-seq from HEK293 cells treated with a control scramble siRNA (wt1 and wt2) or with a Top1-specific siRNA (kd1 and kd2).

***Reviewer #3***:

*Manzo et al. use DRIPc-seq to compare the landscape of R-loops between wild type human cells and cells depleted of Topoisomerase 1 (Top1). They identified two classes of genes that were affected by Top1 depletion, ones that either gain or lose R-loop signal. They observe a correlation of between the regions that gain R-loops with highly transcribed long genes in gene-poor regions, anchored to Laminin B1 domains. In contrast, R-loops depleted regions in Top1 depleted cells correlated with gene-rich regions, rich in replication-initiations regions. The manuscript is written for a sequencing-savvy readership, and as result is quite hard to understand (particularly text describing figures 4-5). This lack of clarity makes the significance of the correlations unclear, consequently also weakening the strength of the conclusions. A more detailed explanation of the experimental design and a better presentation of the results and conclusions in the result section will greatly improve the paper. In general, stating the main conclusion for each paragraph in the results will greatly help the reader understand the manuscript.*

We agree that the manuscript incorporates significant (epi)genomics data and presents a complex dataset. We believe that the topic and the methodologies presented are nonetheless well suited for the undoubtedly sequencing-savvy readership of Genome Biology. Following the three reviewers useful comments we have significantly revised the manuscript and believe that it is much stronger as a result. In particular, we followed this reviewer’s advice and significantly revised the first Results section to present the data in a more direct and approachable manner. We also, when possible, broke down lengthy paragraphs and presented a clear justification for the ideas tested at the beginning and clear conclusions at the end.

*Additional revisions:*

*- Indicate hybrid signal or s9.6 signal in the figure 1b.*

This was added.

*- Change color in S1C and 1c since terminators and promoters are barely distinguishable.*

This was done.

- *The description of the DRIP-seq results in WT vs Top1 depletion is confusing. My understanding is that WT generates 70.000 peaks of hybrids covering around 200MB of genome. Then the authors describe data analysis on the Top1 depletion, concluding that classical peak calling detects differences from WT in just a very small percentages of regions (4%). Analysis of single peaks suggested that the signal in Top1 depleted cells spread from regions in WT. However, these data are not shown in a figure nor is it stated clearly?*

We apologize for any confusion. The reviewer is correct that about 70,000 peaks of signal are called using the traditional peak calling method on WT HEK293 cells and that 4% of these peaks show significant differences between control and Top1-depleted cells. We now provide a screenshot of a typical gene where “spreading” was observed, therefore justifying the development of a more sensitive peak calling algorithm to properly capture the differences. The screenshot can be found in Figure S1E.

*- Then a more sensitive peak calling software (which presumably was used in both WT and Top1 samples?) revealed that gain and loss occurred at 28000 peaks (relative to WT?). It is not clear how those 28000 peaks are just 7% of the total peaks (70000).*

The source of the confusion appears to be a comparison between numbers obtained from the two distinct peak calling methods. Each method produces different numbers and they should only be compared within each method. We have revised this paragraph significantly to make this distinction clearer and now present the data much more directly, with an exact enumeration of peak numbers for gains, losses, compared to total. Supplemental Table 1 presents a full comparison of the results from both methods side by side. We now draw readers in a more forceful way to this important source of information by stating: “see Supplemental Table 1 for a comparison between standard and high-sensitivity methods” (bottom of page 4). We apologize if the previous language was confusing. The reviewer is correct that the high-sensitivity peak calling method was used on all samples. This section of the Results now reads: “To properly account for these events, we optimized a high-sensitivity version of our peak-calling algorithm and applied it to all samples (see Methods). This method identified a total of 399,953 peaks and significant, reproducible R-loop signal gains and losses upon Top1 depletion occurred at 15,112 and 12,977 peaks, respectively (7.02% of total peaks) (Supplemental Fig. S1F, see Supplemental Table 1 for a comparison between standard and high-sensitivity methods)”. We hope this is clear.

*- Also, R-loops in WT cells cover 200Mb. In the top1 depleted cells regions of R-loop gain affected 100MB and loss 36MB (from figure 1C). How are those numbers consistent with Top1 depletion only affecting a small subset of hybrid regions?*

Regions of R-loop gains and loss indeed cover 100 and 36MB of sequence space according to high-sensitivity peak calls. Again, these values cannot be compared to the total value of 200Mb of R-loop space obtained using the standard peak calling algorithm which is far more stringent. As stated above, we appreciate that this may have been confusing. Along with a stronger reference to Supplemental Table 1, we emphasized at the beginning of that paragraph that this analysis concerned high-sensitivity peaks. Overall, only a small portion of total R-loop peaks are affected by Top1 depletion: either 4.07% of a smaller, more stringent set, or 7.02% of a much larger, less stringent set.

*- The authors jump from signal spreading (which presumably means peaks covering more DNA sequence), to increase/decrease of in the region, which is very confusing. Does Top1 depletion make longer hybrid regions, or increase the number of hybrids in a region? These points must be described and discussed more clearly.*

Top1 depletion results in an unexpectedly complex response that involves BOTH increases and decreases in signal intensity which are accompanied by spreading and shrinking of R-loop forming regions compared to control. This complex response is interesting and reveals that Top1 exerts context-dependent regulation on R-loop metabolism – this represents a novel observation compared to simplistic expectations from the literature that Top1 depletion would result in a unidirectional, global increase in R-loops. This is not what is happening. Of course, this in turn poses a challenge to accurately describe a complex response. With all the new edits and data added to the revised manuscript, we believe we went to great lengths to reflect this complexity and to develop suitable methods to quantify and comprehensively describe this interesting dataset. Issues related to signal gains and losses both in terms of intensity and spread were heavily emphasized and the primary focus on the first half of the manuscript, including through several data screenshots (Fig 1E, Fig S1E, Fig S2A,B) and metagene analysis (Fig. 3A, B, C, Fig 4D, and Fig S3B, S4C).

*- The rationale and intro for figure 1E (rDNA analysis) should be presented before the data.*

*This was done; we thank the reviewer for the suggestion.*

*-In figure 4A and 4B and following figures, the authors compare the RLG and RLL with a subset of hybrid controls that match the characteristics of the RLG and RLL regions, but don't show changes in R-loop levels upon Top1 depletion. This subset is different from the one used in the previous figures. It is not clear why the control subset was changed and how the result will look using the previous control subset.*

Before Figure 4, comparisons were limited to “simple” Control (scramble) versus Top1-depleted cells or R-loop losses versus R-loop gains. However, in Figure 4 and 5, we set out to determine if we could identify chromatin features that could specifically predict regions of R-loop gains or loss in response to Top1 depletion. This necessarily involves controlling for as many confounding variables as possible given that chromatin states are highly sensitive to factors such as transcription levels, position, gene size, etc… For this, the approach we took was similar to the one we developed in our 2016 Molecular Cell paper (Ref 4) when we first set out to identify chromatin characteristics of R-loop forming genes. In this particular story, we decided to go one step further and compare the chromatin states of genes undergoing R-loops gains and losses in response to Top1 depletion to the chromatin states of R-loop forming genes matched for expression, position, and length that were not significantly affected by Top1 depletion. Doing so ensured that our findings would be as stringent and relevant as possible. Not doing so is in our minds not scientifically justifiable and would lead to unreliable results. Nonetheless, we significantly revised the text to better justify our approach and cited our prior published work as precedent.

*- There are no arrows in figure 4D as indicated in figure legend. It Is not clear what is the difference between the 3 panels on the right and the 3 on the left.*

The arrows were unexpectedly lost in the conversion from AI to PDF format. We made sure they were kept this time. This should clarify how the three set of panels are different from each other (promoter firing away from LAD at right, towards the LAD at left). We apologize for the inconvenience.

*- Figure 5D terminators are called terminal. Is there a specific reason for this new nomenclature?*

Throughout the manuscript we refer to gene ends (centered around the polyA site) as terminal regions or terminators somewhat interchangeably.

*- The results on the cell cycle delay presented in figure 6 do not appear connected with the regions of gain and loss of R-loops upon Top1 depletion. There is no difference in phenotype between the regions of loss (fig6F) and the control regions used (fig. S6b). How are these results connected with the previous observations? The results in figure 6 do not appear to corroborate or extend the conclusions from the previous figures.*

We believe that the reviewers’ conclusion that the cell cycle delay and replication timing delay we observe is “not connected” to regions of R-loop gains and loss is too strong. As stated in the manuscript, cells “show a trend towards replication delay in a way that appears influenced by, but not strictly dictated by, R-loop gains or losses”. This could be easily explained if for instance replication loss at RLL genes have a long-distance influence on neighboring gene-rich regions that include many “unaffected” genes. Under this scenario, both RLL genes that suffer from Rloop loss directly and their neighbors would show replication delay which is close to what we observe at first glance. A genome-wide analysis of high resolution replication timing data will be necessary to test this hypothesis but this goes beyond the scope of this manuscript.

*- There are numerous typos in the manuscript that need to be corrected*.

The manuscript was curated by an experienced editor.

**Second round of Review**

**Reviewer 1:**

**Please provide your comments for the author(s) on the revised manuscript.**

Manzo et al largely addressed most of my concerns in their revised manuscript. A few issues remain that need to be addressed.

Major Points:

1. Figure 3A is somewhat misleading, as it suggests R-loop signals over RLG genes are substantially higher than over RLL genes except at small genes. Since these plots are not normalized for the number of genes in each group (as far as I can tell), they really are reflecting that more genes over 20 kb are RLG genes than RLL genes, and that the R-loop peaks mainly occur over gene bodies. It is odd to see a metaplot in which the y-axis is the total number of peaks rather than the normalized read density. In my view, this should be changed or at least clarified in the text to indicate the extent to which the high RLG signal for large genes simply reflects more RLG genes > 20 kb relative to RLL genes > 20 kb.

2. Figure 4 and accompanying text is still somewhat confusing and should be fleshed out a bit more. Highly expressed genes are underrepresented within LADs, but the authors show that RLGs are often near LADs and/or H3K9me3 domains while also highly expressed. Are long, highly transcribed genes that are also RLGs more likely to be in close proximity to LADs than long, highly transcribed genes that lack R-loops? The “matched” controls include long, highly transcribed genes with R-loops that do not increase or decrease, but are RLG genes overrepresented near LAD boundaries compared to all genes or all highly expressed genes? In addition, what is the nature of the H3K9me3 peaks within highly transcribed RLG genes? Do these represent one or a few nucleosomes within highly expressed genes marked with H3K9me3 or broader heterochromatin-associated domains within large expressed genes? Perhaps a metaplot of H3K9me3 signal within RLG, RLL, and all genes would show informative differences if this mark plays a role in modulating R-loop levels in the absence of Top1.

Minor Points:

1. Figure 1B: Labels indicating the amount of DNA loaded in each spot should be indicated in the figure.

2. Figure 5E: the correlation coefficient is positive when visual inspection of the data clearly shows a negative correlation. How can this be?

**Evaluation. Has the author satisfactorily responded to your previous review?**

Yes

**Reviewer 2:**

**Please provide your comments for the author(s) on the revised manuscript.**

The authors have adequately addressed my concerns.

**Evaluation. Has the author satisfactorily responded to your previous review?**

Yes

**Reviewer 3:**

**Please provide your comments for the author(s) on the revised manuscript.**

The manuscript has been revised to address all previous concerns and will make an excellent contribution to the literature.

**Evaluation. Has the author satisfactorily responded to your previous review?**

Yes

**Authors’ response to reviewer 1:**

*Figure 3A is somewhat misleading, as it suggests R-loop signals over RLG genes are substantially higher than over RLL genes except at small genes. Since these plots are not normalized for the number of genes in each group (as far as I can tell), they really are reflecting that more genes over 20 kb are RLG genes than RLL genes, and that the R-loop peaks mainly occur over gene bodies. It is odd to see a metaplot in which the y-axis is the total number of peaks rather than the normalized read density. In my view, this should be changed or at least clarified in the text to indicate the extent to which the high RLG signal for large genes simply reflects more RLG genes > 20 kb relative to RLL genes > 20 kb.*

We have now normalized the metaplots per gene in each length category. Figure 3A was replaced and the figure legend was updated to indicate this. The conclusions are unchanged and the graphs themselves are only slightly modified (save for the y-axis).

*Figure 5E: the correlation coefficient is positive when visual inspection of the data clearly shows a negative correlation. How can this be?*

This was a simple typo for which we apologize. The correlation coefficient is -0.95, not 0.95. The figure was edited.
